# Peptoid-based antimicrobial strategies against polymyxin-resistant Gram-negative bacteria

**DOI:** 10.1093/jambio/lxag093

**Published:** 2026-04-11

**Authors:** Shyam Kumar Mishra, Jiawei Shen, Tanzina Akter, Abiye Tigabu, Umme Laila Urmi, Meseret Alem Damtie, Elias Shiferaw Mekonen, Rajesh Kuppusamy, Kristian Sørensen, Jennifer S Lin, Edgar H H Wong, Alex Hui, Annelise E Barron, Mark Willcox

**Affiliations:** School of Optometry and Vision Science, Faculty of Medicine and Health, University of New South Wales, Sydney, NSW 2052, Australia; Department of Microbiology, Maharajgunj Medical Campus, Institute of Medicine, Tribhuvan University, Kathmandu 44600, Nepal; School of Optometry and Vision Science, Faculty of Medicine and Health, University of New South Wales, Sydney, NSW 2052, Australia; School of Optometry and Vision Science, Faculty of Medicine and Health, University of New South Wales, Sydney, NSW 2052, Australia; Microbial Biotechnology Division, National Institute of Biotechnology, Dhaka 1349, Bangladesh; School of Optometry and Vision Science, Faculty of Medicine and Health, University of New South Wales, Sydney, NSW 2052, Australia; School of Optometry and Vision Science, Faculty of Medicine and Health, University of New South Wales, Sydney, NSW 2052, Australia; School of Optometry and Vision Science, Faculty of Medicine and Health, University of New South Wales, Sydney, NSW 2052, Australia; School of Optometry and Vision Science, Faculty of Medicine and Health, University of New South Wales, Sydney, NSW 2052, Australia; School of Optometry and Vision Science, Faculty of Medicine and Health, University of New South Wales, Sydney, NSW 2052, Australia; School of Chemistry, Faculty of Science, University of New South Wales, Sydney, NSW 2052, Australia; Department of Bioengineering, School of Medicine and School of Engineering, Stanford University, Stanford, CA 94305, USA; Department of Bioengineering, School of Medicine and School of Engineering, Stanford University, Stanford, CA 94305, USA; School of Chemical Engineering, Faculty of Engineering, University of New South Wales, Sydney, NSW 2052, Australia; School of Optometry and Vision Science, Faculty of Medicine and Health, University of New South Wales, Sydney, NSW 2052, Australia; School of Optometry and Vision Science, University of Waterloo, Waterloo, ON N2L 3G1, Canada; Department of Bioengineering, School of Medicine and School of Engineering, Stanford University, Stanford, CA 94305, USA; School of Optometry and Vision Science, Faculty of Medicine and Health, University of New South Wales, Sydney, NSW 2052, Australia

**Keywords:** antimicrobial peptoid, antimicrobial resistance, peptidomimetic, polymyxin, synergy

## Abstract

**Aims:**

Polymyxins remain the mainstay antibiotic for the treatment of infections caused by multidrug-resistant bacteria. However, with the increase in the use of polymyxins, the simultaneous rise of polymyxin-resistance cases has been another global threat, necessitating the need for novel therapeutic strategies. This work aimed to evaluate the antibacterial activity of cationic peptoids against polymyxin-resistant bacteria of global priority.

**Methods and results:**

Three paired polymyxin-sensitive/polymyxin-resistant strains were included, along with two clinical isolates and one reference strain. Out of nine cationic peptoids, TM8 showed the most potent activity against the polymyxin-resistant bacteria with a geometric mean minimum inhibitory concentration (MIC) of 15.6 μg mL^−1^. The MIC of TM8 was 2-fold higher in polymyxin-resistant cases. TM8 synergized with colistin, rifampicin, and ciprofloxacin in polymyxin-resistant bacteria with reductions in MIC of antibiotics ranging from 8- to 64-fold. *Enterobacter cloacae* did not develop resistance to TM8 upon repeated subpassage at its sub-MIC, whereas it evolved to resist ciprofloxacin by sixty-four-fold under the same conditions. A concentration-dependent membrane-disruptive potential activity was noted in flow cytometry using live-dead staining. The impact of monovalent cations was small (≤2-fold change), while in the presence of divalent cations, the MIC of TM8 increased up to 4-fold.

**Conclusion:**

This study presents TM8 as a potential candidate antimicrobial against polymyxin-resistant bacteria. Further studies are recommended focusing on safety, pharmacokinetics, and pharmacodynamics of this compound.

Impact statement This study demonstrates the potential of peptoid-based antimicrobials, particularly TM8, as a promising alternative against polymyxin-resistant Gram-negative pathogens. The findings highlight their synergistic or additive activity with existing antibiotics, including colistin, and their resilience against resistance development. These results provide a pathway toward novel therapeutic strategies to combat antimicrobial resistance.

## Introduction

Carbapenem-resistant *Enterobacterales*, including *Klebsiella pneumoniae, Escherichia coli, Enterobacter cloacae, Acinetobacter baumannii*, and *Pseudomonas aeruginosa*, are listed as threat pathogens by both the United States Centers for Disease Control and Prevention (CDC) and the World Health Organization (WHO) (CDC 2024, WHO 2024). For the treatment of infections caused by such carbapenem-resistant and other multidrug-resistant (MDR) Gram-negative bacteria, polymyxins (polymyxin B and colistin) are often used as the last-resort antibiotics (Velkov et al. 2013, Poirel et al. 2017, Nang et al. 2021). However, their increased use in clinical settings and animal husbandry has led to an alarming increase in polymyxin-resistant infections, thereby pushing back toward a “pre-antibiotic era,” where effective treatment options for such infections are severely limited (Lee et al. 2009, Rojas et al. 2017, Li et al. 2019, Van Der Kolk et al. 2019, Jiang et al. 2025). Consequently, pandrug-resistant (PDR) *K. pneumoniae, A. baumannii*, and *P. aeruginosa* infections are not uncommon in clinical settings (Valencia et al. 2009, de Man et al. 2018, Nishida and Ono 2020, Ferry et al. 2022). There are only limited antibiotics in the pipeline active against Gram-negative bacteria. Therefore, development of new antimicrobials has become an immediate priority to reduce the estimated 11.1 million (95% UI 9.08–13.2 million) deaths that will be caused by MDR Gram-negative bacteria by 2050 (Naghavi et al. 2024).

The Gram-negative bacterial cell envelope consists of an inner cytoplasmic membrane, a thin peptidoglycan layer, and an asymmetric outer membrane (OM). This OM is typically composed of an inner leaflet of phospholipids, with lipopolysaccharides (LPS) as a main lipid in the outer leaflet. LPS consists of lipid A, an oligosaccharide core, and the O antigen (Maher and Hassan 2023). As OMs are generally impermeable in nature, Gram-negative bacteria are challenging to treat (O’Leary et al. 2022). Polymyxins target the OM as their primary bactericidal mechanism (Trimble et al. 2016, Poirel et al. 2017). However, Gram-negative bacteria can become resistant to polymyxins through various mechanisms. These include innate resistance, mutations in genes involved in LPS synthesis, adaptative responses, or acquisition of plasmid-mediated mobile colistin resistance-1 (*mcr-1*) or its variants (El-Sayed Ahmed et al. 2020), with mechanisms varying across species and strain (Falagas et al. 2010). Recently, there has been an increase in the global spread of *mcr*-positive *K. pneumoniae* with reports from at least 57 countries (Liu et al. 2024), and such resistant variants due to *mcr* genes are more problematic than chromosomally acquired mutations (Liu et al. 2016).

Antimicrobial peptides (AMPs) are promising therapeutic alternatives against MDR bacteria (Xuan et al. 2023). However, due to their shared target of the OM, both polymyxins and AMPs can be affected by similar resistance mechanisms (Bengoechea and Sa Pessoa 2019). Exposure of *K. pneumoniae* to polymyxin has been found to induce resistance to both polymyxin and AMPs by upregulation of genes responsible for capsule polysaccharide and lipid A modification (Llobet et al. 2011). Despite this, many AMPs are still active against *mcr*-positive bacteria (Siedentop et al. 2024). However, challenges such as short half-life, potential cytotoxicity, and high cost of synthesis are limiting factors for the clinical utilization of AMPs (Mishra et al. 2025a). These shortcomings are addressed by mimetics of AMPs, including peptoids, which are synthetic oligomers of *N*-substituted glycine units, mimicking peptide structures, with broad range of activity (Sierra and Vinas 2021).

The antimicrobial activity of selected peptoids against ESKAPEE pathogens has been shown, with particular emphasis on one peptoid, TM8, and its efficacy against carbapenemases-metallo-β-lactamase (MBL)-producing pathogens (Mishra et al. 2025b). In the current study, the effectiveness of TM8 against polymyxin-resistant Gram-negative members of the ESKAPEE group is reported. The test panel included clinical isolates, a reference strain, representative wild-type strains, and their laboratory-derived colistin-resistant mutants.

## Materials and methods

### Antimicrobial peptidomimetics and antibiotics used in this study

A previously described standard submonomer solid-phase synthesis approach was used to synthesize the peptoids (final purity > 97%), as it offers control over sequence, chain length, and side-chain modifications (Mishra et al. 2025b, Nielsen et al. 2022, Jenssen 2025). Melimine and Mel4 peptides (final purity > 90%) were purchased from Auspep Peptide Company (Tullamarine, Victoria, Australia). Melimine is an arginine-rich cationic hybrid peptide of melittin and protamine (Willcox et al. 2008), while Mel4 is a shorter sequence of melimine lacking tryptophan, leucine, and isoleucine that are otherwise present in melimine.

**Figure 1:**
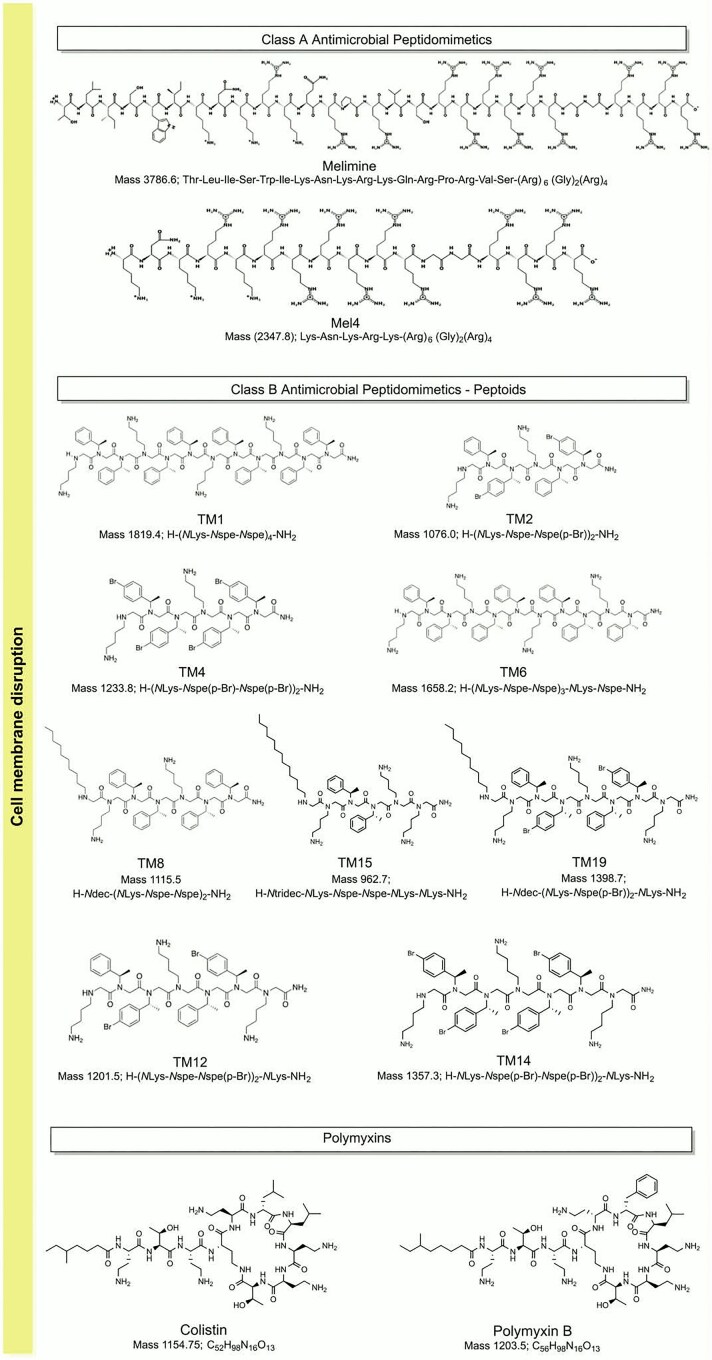
Antimicrobials used.

Ciprofloxacin, gentamicin, minocycline hydrochloride, chloramphenicol, ceftazidime, colistin, polymyxin B, and rifampicin were purchased from Sigma-Aldrich, St. Louis, MO, USA. Similarly, cefotaxime and meropenem trihydrate (USP reference standard) were obtained from Cayman Chemical, Ann Arbor, Michigan, USA, and Rockville, MD, USA, respectively. These antibiotics were dissolved in solvents and stored as recommended by Clinical and Laboratory Standards Institute (CLSI) (CLSI 2025), or according to the product leaflet. The structures of polymyxins and antimicrobial peptidomimetics, including peptoids are shown in Fig. 1.

### Antimicrobial sample preparation

Stock solutions of melimine, Mel4, and peptoids were prepared in 1× phosphate buffered saline (PBS: NaCl 8 g L^−1^, KCl 0.2 g L^−1^, Na_2_HPO_4_ 1.15 g L^−1^, KH_2_PO_4_ 0.2 g L^−1^, without divalent cations, pH 7.2) at a final concentration of 2 mg/mL in polypropylene microcentrifuge tubes, and were stored at -80°C. The antimicrobial stock solutions were filter-sterilized using 0.22 µm-size membrane filters (Millex^®^-GP, Merck Millipore Ltd., Tullagreen, Carrigtwohill, Co. Cork, Ireland) and aliquoted to avoid repeated freeze-thaw cycles. Working solutions of all antimicrobials were prepared by diluting the stock solutions in Mueller Hinton Broth (MHB; Oxoid, Basingstoke, UK).

### Bacterial isolates

Polymyxin-resistant bacterial strains belonging to the WHO critical priority pathogens were selected (Table 1), including paired wild-type and resistant strains of *A. baumannii* ATCC 19606, *K. pneumoniae* ATCC 13883, and *P. aeruginosa* PAO1. Resistance had been previously induced by serial passaging or plating on colistin 10 µg mL^−1^ (Moffatt et al. 2010, Velkov et al. 2014, Jiang et al. 2022). Two additional resistant clinical isolates, *P. aeruginosa* 216 and *E. cloacae* 008 along with a reference strain of *E. coli* NCTC 13846, were also included.

**Table 1:** List of microbes used in this study.

Bacteria	Strain number	Known resistance mechanism or associated resistance gene	Clinical specimen	Reference
*A. baumannii*	ATCC 19606	*sul*2, *bla*_OXA-98_, *bla*_ADC_	Urine	Moffatt et al. (2010), Hamidian et al. (2020), Zhu et al. (2020)
*A. baumannii*	19606-R	Resistance to colistin due to loss of outer membrane	−	Moffatt et al. (2010)
*E. cloacae*	008	Not known	Contact lens	−
*E. coli*	NCTC 13846	*mcr-1, bla*_TEM-1_, *bla*_CTX-M-27_, *aadA1, aadA2, aac(3)-IIa, lnuF, mph*(A), *dfrA14, tet*(A), *sul1, sul3, floR*	Blood	Akhoundsadegh et al. (2019)
*K. pneumoniae*	ATCC 13883	*bla*_SHV-1_	Not reported	Velkov et al. (2014)
*K. pneumoniae*	13883-R	This paper	−	Velkov et al. (2014)
*P. aeruginosa*	PAO1	*aph(3′)-IIb, bla*_PAO_, *bla*_OXA-50_, *fosA, catB7*	Wound*	Jiang et al. (2022), Mahamad Maifiah et al. (2022)
*P. aeruginosa*	PAO1-R	This paper	−	Jiang et al. (2022)
*P. aeruginosa*	216	*aph(3′)-IIb, crpP, bla*_OXA-50_ *, fosA, catB*	Cornea	Khan et al. (2020)

*sul*2, sulfonamide resistant dihydropteroate synthase*; bla*_OXA_, oxacillinase; *bla*_ADC_, *Acinetobacter*-derived cephalosporinase; *mcr-1*, mobilized colistin resistance-1; *bla*_TEM_, Temoniera; *bla*_CTX-M-15_, cefotaximase-munich β-lactamase; *aad*, aminoglycoside adenyltransferase; *aac*, aminoglycoside N-acetyltransferase; *lnuF*, lincosamide nucleotidyltransferase; *mph*(A), macrolide 2′-phosphotransferase I; *dfr*, dihydrofolate reductase; *tet*, tetracycline efflux pump; *floR*, florfenicol resistance gene; *bla*_SHV_, sulfhydryl variable; *aph*, aminoglycoside phosphotransferase; *fosA*, fosfomycin resistance gene; *catB*, chloramphenicol acetyltransferase B; *crpP*, ciprofloxacin resistance protein. *PAO1 strain originated by spontaneous mutation of the PAO isolate, originally isolated from a wound specimen in Melbourne, Australia.

### Bacterial suspension preparation

Bacteria were cultured overnight at 37°C in Trypticase soy broth (TSB; Oxoid, Basingstoke, UK). Cells were washed twice with 1 × PBS and resuspended in Mueller-Hinton broth (MHB; Oxoid, UK) at approximately 1.5 × 10^8^ CFU mL^−1^. This suspension was subsequently adjusted to 10^6^ CFU mL^−1^ for all the assays, except for the antimicrobial synergy test, in which a concentration of 5 × 10^6^ CFU mL^−1^ was used (Mishra et al. 2025b).

### Minimum inhibitory concentration determination

Minimum inhibitory concentrations (MICs) were determined by the broth microdilution, as previously described (Mishra et al. 2025b, CLSI 2025). Briefly, antimicrobials were serially twofold diluted in 96-well polystyrene plates (Costar, Sigma-Aldrich, USA) containing 100 µL of MHB per well, except for the growth control and sterility control (blank) wells. Subsequently, 100 µL of the bacterial inoculum prepared in MHB was added to each well, except for the blank wells, to achieve a final inoculum of approximately 5 × 10^5^ CFU mL^−1^. The blank wells contained only MHB, while the growth control wells contained the bacterial suspension without antimicrobials. Plates were sealed with cling film to minimize evaporation and incubated at 37°C with shaking at 120 rpm for 18–20 hours. The MIC was defined as the lowest concentration of the antimicrobial resulting in ≥90% reduction in bacterial density based on viable plate counts. Susceptibility was interpreted using CLSI chart (CLSI 2025). All assays were performed in triplicate with two independent biological replicates.

### Nucleic acid extraction and whole genome sequencing

As the mechanisms of polymyxin resistance for *K. pneumoniae* 13883-R and PAO1-R strains were not known, genomic DNA was extracted from overnight cultures using a DNeasy Blood & Tissue Kit (Qiagen, Hilden, Germany). The concentration and integrity of the extracted DNA were assessed using the Qubit 3.0 Fluorometer (Thermo Fisher Scientific, Wilmington, USA) and 1% agarose gel electrophoresis (Tanon, Shanghai, China), respectively. The library was constructed using the VAHTS Universal Plus DNA Library Prep Kit for Illumina (ND617-02, Vazyme, Nanjing, China) by randomly shearing genomic DNA into short fragments. The obtained fragments were end-repaired, A-tailed, and ligated to Illumina adapters. The adapter-ligated fragments were amplified using polymerase chain reaction (PCR), followed by size selection and purification using magnetic beads. The library quality was then assessed using the QSep-400 (BiOptic, Taipei, China) and Qubit 3.0 Fluorometer (Thermo Fisher Scientific, Wilmington, USA). Finally, the quantified libraries were barcoded, pooled, and sequenced at 150 bp paired-end on the Illumina NovaSeq X platform (Illumina, San Diego, USA).

### Bioinformatics analysis

Raw sequencing data underwent quality control (QC) using FastQC to ensure accuracy and reliability. Incorrect bases, adapter-containing reads, and low-quality reads were trimmed using Trimmomatic version 0.39. Samples that passed QC were assembled using MEGAHIT v1.2.9, and genome annotation was performed using Prokka version 1.14.6. To determine the antimicrobial resistance (AMR) gene profile, the whole genome sequencings (WGSs of *K. pneumoniae* 13883-R (BioSample: SAMN48355430), PAO1-R (BioSample: SAMN48355431), and published PA216 (BioProject: PRJNA590804, BioSample: SAMN13340390) (Khan et al. 2020) were blasted against the Comprehensive Antibiotic Resistance Database (CARD), with genes associated with “peptide antibiotic” being examined. Additionally, acquired resistance genes were identified using ResFinder version 4.7.2. The AMR profiles of the resistant strains (*K. pneumoniae* 13883-R and PAO1-R) are presented in supplementary file 1 (Tables S1, S2, S3, and S4).

### Variant identification and functional annotation

The variants between genomes of wild-type and mutant strains were identified using Snippy (v4.6.0), a bacterial variant-calling pipeline. Functional annotation of identified variants was performed using SnpEff (v5.0e). Genes were classified as high-impact by SnpEff if they contained at least one variant having a high impact on gene function, and were included. The full list of high-impact variants of resistant strains (*K. pneumoniae* 13883-R and PAO1-R) based on SnpEff are presented in supplementary file 1 (Tables S5 and S6).

### Ability of *Enterobacter cloacae* to develop resistance to antimicrobials

The ability of *E. cloacae* 008 to become resistant to melimine, TM8, or ciprofloxacin was determined by serial passage in sub-inhibitory concentrations (0.5 × MIC) over 15 days, as described previously (Willcox et al. 2008). This strain was selected as it had not been previously tested to determine whether bacteria could become resistant to the AMPs or peptoids. Cultures (10^6^ CFU/mL) were passaged daily, with MICs determined each day. If an increase was observed, the antimicrobial concentration was adjusted to maintain 0.5 × MIC. Daily purity checks were performed to rule out potential cross-contamination. Any increase in MIC implied potential for the bacteria to acquire resistance to the tested antimicrobial agent.

### Antimicrobial synergy

To determine the *in vitro* efficacy of TM8 in combination with selected antibiotics, a checkerboard microdilution test was performed, and the fractional inhibitory concentration (FIC) of the antimicrobials was calculated. The FIC index was then determined to assess whether the combinations yielded synergistic, antagonistic, or indifferent effects, as described previously (Mishra et al. 2025b), against colistin-resistant bacteria. TM8 and different antibiotics (colistin, minocycline, rifampicin, ciprofloxacin, and gentamicin), were first diluted in two different 96-well microtiter plates in a serial fashion along orthogonal direction. These two-dilution series were then combined in a single 96-well microtiter plate to create a two-dimensional concentration gradient of the antimicrobial combinations. Following this, a 10 µL aliquot of bacterial suspension (5 × 10^6^ CFU mL^−1^) was added to each well, except for the sterility control. The first column contained only antibiotics, while the first row received only TM8 at various concentrations so that they could both provide the MICs of the tested antimicrobials alone, in the same plate, which affirms the previously determined MIC results and facilitates FIC calculation. Sterility control wells contained a total of 110 µL of MHB, whereas growth control wells contained 110 µL of bacterial inoculum with no antimicrobials. The plates were then incubated at 35 ± 2°C for 18–24 hours. FIC values for each antimicrobial combination were calculated as follows:


\begin{eqnarray*}{\mathrm{FIC\ of\ TM8 }}\left( {{\mathrm{FI}}{{{\mathrm{C}}}_{{\mathrm{TM8}}}}} \right){\mathrm{ = MIC\ of\ TM8\ in\ combination/MIC\ of\ TM8\ alone}}\end{eqnarray*}

\begin{eqnarray*}
{\mathrm{FIC\ of\ antibiotic }}\left( {{\mathrm{FI}}{{{\mathrm{C}}}_{{\mathrm{Antibiotic}}}}} \right)= {\mathrm{MIC\ of\ antibiotic\ in\ combination/MIC\ of\ antibiotic\ alone}}
\end{eqnarray*}

The FIC index (ƩFIC) was determined using the formula: ƩFIC = FIC_TM8_ + FIC_Antibiotic_. Interpretation of the FIC index was as follows: synergy for ƩFIC ≤ 0.5; additive for 0.5 < ƩFIC ≤ 1; indifference for 1 > ƩFIC ≤ 4; and antagonism for ƩFIC > 4 (Odds 2003). At least two independent assays were performed, each with a minimum of two technical replicates.

### Assay for membrane permeability of TM8

The membrane-permeabilizing ability of TM8 was tested against *A. baumannii* ATCC 19606 and *A. baumannii* 19606-R using flow cytometry. Both strains, adjusted to approximately 10^6^ CFU mL^−1^, were treated for 2 hours at 1 × MIC and 4 × MIC, following a previously described method, with simultaneous staining using SYTO 9 (7.5 µmol L^−1^) and propidium iodide (PI) (30 µmol L^−1^) (Invitrogen^TM^, LIVE/DEAD^TM^ BacLight Bacterial Viability Kit, Eugene, Oregon, USA) for 15 minutes at room temperature in the dark (Mishra et al. 2025b). Unstained and single-stained controls (SYTO 9-only and PI-only) were included for fluorescence compensation and gating. Data were acquired on a BD FACSymphony^TM^ A3 (BD Biosciences) flow cytometer operated with BD FACSDiva v9.3. Samples were acquired at a low flow rate (12 µL minute^−1^), with a minimum of 30 000 events recorded per sample. SYTO 9 fluorescence was excited with the 488 nm blue laser and detected using B515-A detector (515/20 nm bandpass filter), whereas PI fluorescence was excited with the 561 nm yellow-green laser and detected using the YG610-A detector (610/20 nm bandpass filter). Single-stained controls were used for compensation. Forward scatter and side scatter were recorded in logarithmic mode for populating gating.

### Effect of salts on the antimicrobial activity of TM8

To gain additional insight into the influence of salts that may be encountered in serum or other body fluids, the MIC of TM8 in the presence of different salts was studied. For MIC, the same procedure as described above was followed, but different concentrations of salts were used in the MHB (Cashman-Kadri et al. 2023). The impact of monovalent cations was tested using 150 mmol L^−1^ sodium chloride (Sigma-Aldrich, USA), 4.5 mmol L^−1^ potassium chloride (Univar, Ajax Finechem, NSW, Australia), and 6 µmol L^−1^ ammonium chloride (ChemSupply, Gillman SA, Australia). Similarly, 1 mmol L^−1^ magnesium chloride (ChemSupply, Gillman SA, Australia), and 2.5 µmol L^−1^ calcium chloride (Unilab, Ajax Finechem, NSW, Australia) were included to examine the effect of divalent cations on MIC. In all cases, salts were added after dilution of TM8 in MHB, and osmolarity effects were controlled by preparing corresponding salt-supplemented controls without TM8.

### Statistical analysis

To evaluate the effects of treatment (TM8, ciprofloxacin, and melimine), time, and their interaction on MIC fold-change, a two-way ANOVA was performed followed by Dunnett’s post hoc test. The Greenhouse-Geisser correction was applied to adjust for any violations of the sphericity assumption. Results are presented as mean ± standard error of the mean (SEM), and statistical significance was defined as *P* < 0.05. GraphPad Prism (version 10.0.3, GraphPad Software, San Diego, CA, USA) was used for data analysis.

## Results

### High-impact mutations in polymyxin resistance-related genes

Based on functional annotation, there were differences in single nucleotide polymorphisms of 58 high-impact genes in *K. pneumoniae* and seven in *P. aeruginosa* polymyxin-resistant strains when compared with their wild-type strains. Among these, genes associated with polymyxin resistance are presented in Table 2. A comprehensive list of all high-impact genes is provided in Supplementary file S1 (Tables S5 and S6 for *K. pneumoniae* and *P. aeruginosa*, respectively). For *K. pneumoniae* and *P. aeruginosa* strains, no acquired resistance genes for polymyxin resistance were identified in the ResFinder database. Those genes associated with peptide resistance in CARD are shown in Table 2, and include insertion of stop codons or, for example, single nucleotide polymorphisms that resulted in amino acid changes to ArnA and NalC.

**Table 2:** Polymyxin resistance-associated high-impact genes in *K. pneumoniae* and *P. aeruginosa* mutants, and in a clinical isolate of *P. aeruginosa*.

Bacteria	Gene	Variant effect	Predicted effect	Function associated with AMR
*K. pneumoniae* 13883-R	*evgA*	Stop gained	Tyr87*	Part of the two-component system EvgSA, regulates multidrug resistance (Lai et al. 2003, Ramos et al. 2016, Zhang and Wang 2023).
	*gltP*	Stop gained	Gln437*	Related to phospholipid transport in the membrane, mutations can lead to tolerance to AMPs (Sandin et al. 2022).
	*ccrB*	Stop gained	Tyr241*	Associated with resistance to colistin via lipid A modification with 4-amino-4-deoxy-L-arabinose (L-Ara4N) and palmitoylation (Sun et al. 2020, Sanchez-Leon et al. 2023).
	*mlaB*	Stop gained	Ser96*	May be involved in polymyxin resistance via its phospholipid trafficking across the bacterial envelope to maintain outer membrane integrity (Liu et al. 2021, Sun et al. 2022).
	*lptD*	Amino acid change (Missense SNP)	Ser45Gly	A component of the LptD/LptE transmembrane ABC transporter involved in the transport of LPS across the inner membrane (Janssen et al. 2020).
*P. aeruginosa* PAO1-R	*pmrA*	Frameshift variant	Glu163fs	The response regulator of the PmrAB two-component regulatory system, regulates resistance to colistin and polymyxin (Gunn et al. 1998, Jafari-Ramedani et al. 2024).
	*mexT*	Frameshift variant	Gln80fs	A transcriptional regulator, a major regulator of multidrug resistance and virulence. Activates the *mexEF-oprN* efflux pump.
	*masA*	Frameshift variant	Ser219fs	Encodes Enolase phosphatase E-1; no direct function associated with AMR (https://pseudomonas.com/feature/show?id=106140).
	*napA*	Frameshift variant	Phe11fs	Encodes a periplasmic nitrate reductase.
	PA0683	Frameshift variant	Val73fs	HxcY (probable type II secretion system protein).
	PA1327	Frameshift variant	Pro642fs	Probable protease (https://pseudomonas.com/feature/show?id=105422); No direct function associated with AMR.
	PA2141	Frameshift variant	Leu173fs	Hypothetical protein (https://pseudomonas.com/feature/show?id=107062).
*P. aeruginosa* 216	*arnA*	Amino acid change (Missense SNP)	Cys312Ser; Ser313Gly	ArnA is involved in the addition of 4-amino-4-deoxy-L-arabinose to lipid A of LPS (Gatzeva-Topalova et al. 2005).
	*nalC*	Amino acid change (Missense SNP)	Gly71Glu; Ala186Thr	Mutations associated with over expression of MexAB-OprM (Braz et al. 2016).

SNP = Single Nucleotide Polymorphism; fs = frameshift, e.g. Glu16fs indicates frameshift mutation starting at residue 297; *=stop codon, e.g. Tyr87* means mutation introducing a stop codon at tyrosine 87. A SNP was also found in *mexB* (Thr89Ile) of *P. aeruginosa* 216, but this was also present in the parent (and resistant) PAO1 strains and so reasoned to be not involved in polymyxin resistance.

### MIC of antibiotics against different Gram-negative bacterial strains

The average MICs of different classes of antibiotics against polymyxin-resistant bacteria are presented in Table 3. Compared to their wild-type strains, the laboratory-induced colistin-resistant mutants had a 128-fold higher MICs of polymyxin B and colistin. However, there was no appreciable difference in the MIC values of other antibiotics, except in case of *A. baumannii*, which in general had increased resistance to most antibiotics.

**Table 3:** MIC of antibiotics against bacterial isolates.

	MIC of antibiotics (µg mL^−1^)									
Gram-negative strains	Amino-glycoside	Tetracycline	Phenicol	Extended-spectrum cephalosporins		Carbapenem	Polymyxins		Fluoro-quinolone	Ansamycin
	Gen	Mino	Chl	Cefot	Ceftaz	Mer	Col	PmB	Cip	Rif
***A. baumannii* ATCC 19606**	4	ND	NR	8	8	0.5	2	2	1	ND
***A. baumannii* 19606-R**	256	4	NR	>512	>512	0.5	256	256	256	32
***E. cloacae* 008**	16	8	≥32	1	4	1	32	32	0.25	ND
***E. coli* NCTC13846**	1	8	8	0.5	1	1	8	4	>256	16
***K. pneumoniae* ATCC 13883**	≤0.25	1	2	2	2	2	2	2	≤0.25	16
***K. pneumoniae* 13883-R**	≤0.25	2	8	2	2	2	256	256	≤0.25	16
***P. aeruginosa* PAO1**	NR	NR	NR	NR	8	ND	1	1	0.5	NR
***P. aeruginosa* PAO1-R**	NR	NR	NR	NR	8	ND	128	128	≤0.25	NR
***P. aeruginosa* 216**	NR	NR	NR	NR	2	1	16	16	0.5	NR

ND: Not determined; NR: Not recommended by CLSI 2025, therefore, not tested; Gen = Gentamicin; Mino = Minocycline; Chl = Chloramphenicol; Cefot = Cefotaxime; Ceftazidime; Mer = Meropenem; Col = Colistin; PmB = Polymyxin B; Cip = Ciprofloxacin; Rif = Rifampicin.

### MIC of antimicrobial peptoids along with melimine and Mel4

Most of the paired polymyxin-resistant/susceptible strains had a ≤ 2-fold difference in MIC to melimine, Mel4, or the peptoids. The exception was for the *A. baumannii* pairing with melimine, TM1, TM2, and TM6 (Table 4). TM8 had the lowest MIC range against all the polymyxin-resistant bacteria, with only a 2-fold difference in the MIC of TM8 compared to their susceptible forms, and an overall geometric mean MIC of 15.6 µg mL^−1^. The MIC fold-change in wild-type and mutant strains for melimine, Mel4, and peptoids is illustrated in Supplementary Fig. S1. For polymyxin-resistant clinical isolates (*E. cloacae* and *P. aeruginosa* 216) and the reference strain (*E. coli* NCTC 13846), TM1, TM4, TM8, and TM15 had MICs ≤ 15.6 µg mL^−1^.

**Table 4:** MIC of antimicrobial peptidomimetics against ESKAPEE pathogens.

	MIC of peptidomimetics (µg mL^−1^)										
Strains	Melimine	Mel4	TM1	TM2	TM4	TM6	TM8	TM12	TM14	TM15	TM19
*A. baumannii* ATCC 19606	62.5	125	3.9	31.2	15.6	7.8	3.9	125	31.2	62.5	7.8
*A. baumannii* 19606-R	250	250	125	125	7.8	125	7.8	125	15.6	125	15.6
*E. cloacae* 008	500	125	15.6	125	15.6	62.5	15.6	125	31.2	15.6	31.2
*E. coli* NCTC 13846	125	125	7.8	31.2	7.8	7.8	7.8	15.6	15.6	15.6	15.6
*K. pneumoniae* ATCC 13866	31.2	62.5	62.5	125	62.5	125	31.2	125	125	125	125
*K. pneumoniae* 13866-R	31.2	62.5	62.5	125	62.5	125	62.5	125	125	125	125
*P. aeruginosa* O1	250	250	62.5	125	62.5	15.6	15.6	62.5	62.5	31.2	15.6
*P. aeruginosa* O1-R	250	500	125	250	125	31.2	31.2	125	125	31.2	15.6
*P. aeruginosa* 216	500	250	15.6	15.6	7.8	15.6	7.8	31.2	15.6	15.6	7.8
Geometric mean MIC for polymyxin-resistant bacteria	198.5	176.8	35.0	78.4	19.6	39.3	15.6	70.1	35.0	35.0	22.1

### Live-dead cell assay of TM8-treated paired wild-type and mutant strains of *A. baumannii* ATCC 19606

**Figure 2:**
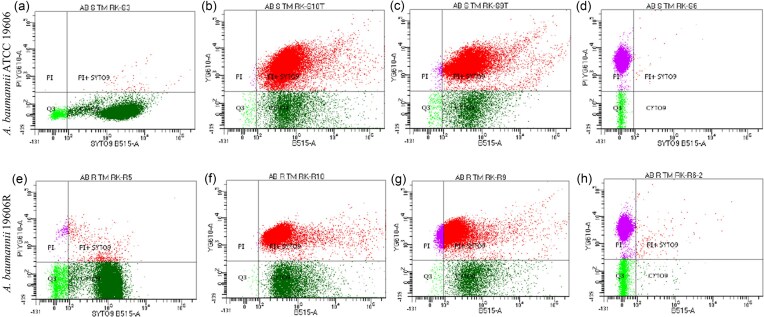
Membrane permeabilization of *A. baumannii* ATCC 19606 (Fig. 2a-d) and *A. baumannii* 19606-R (Fig. 2e-h) determined by flow cytometry with SYTO 9 and propidium iodide staining on treatment with 1 × PBS (negative control) (a and e), TM8 at their respective 1 × MIC (b and f), 4 × MIC (c and g), and 1% Triton X-100 (positive control) (d and h).

As TM8 demonstrated the strongest antimicrobial activity against polymyxin-resistant bacteria, it was further tested on *A. baumannii* ATCC 19606 and its polymyxin B-resistant mutant, as representative bacteria, to evaluate its membrane-disruptive potential. A live-dead cell assay, assessed by flow cytometry, was performed to determine bacterial viability in the presence of TM8. At 1 × MIC, the proportion of live cells decreased from 95.2% to 19.5% in the ATCC 19606 and from 90.2% to 28.2% in the resistant mutant (Table 5). At 4 × MIC, there were more dead cells in the mutant as compared to the ATCC 19606 strain (Figs 2c and g).

**Table 5:** Populations of *A. baumannii* ATCC 19606 wild-type and colistin-resistant mutant showing different staining patterns under different treatment conditions.

		% of bacteria			
Strains	Condition	Unstained	SYTO 9-stained	PI-stained	Mixed population
*A. baumannii* ATCC 19606	Control	4.5	95.2	0	0.3
	1 × MIC	0.6	19.6	0.1	79.8
	4 × MIC	1.1	15.7	0.7	82.5
	1% Triton X-100	3	0	96.3	0.1
*A. baumannii* 19606-R	Control	6.9	90.2	0.6	2.2
	1 × MIC	0.1	28.2	0.1	71.6
	4 × MIC	0.4	16.9	11.1	71.5
	1% Triton X-100	10.4	0.3	88.8	0.6

### Ability of a polymyxin-resistant bacterium to develop resistance to peptoid

**Figure 3:**
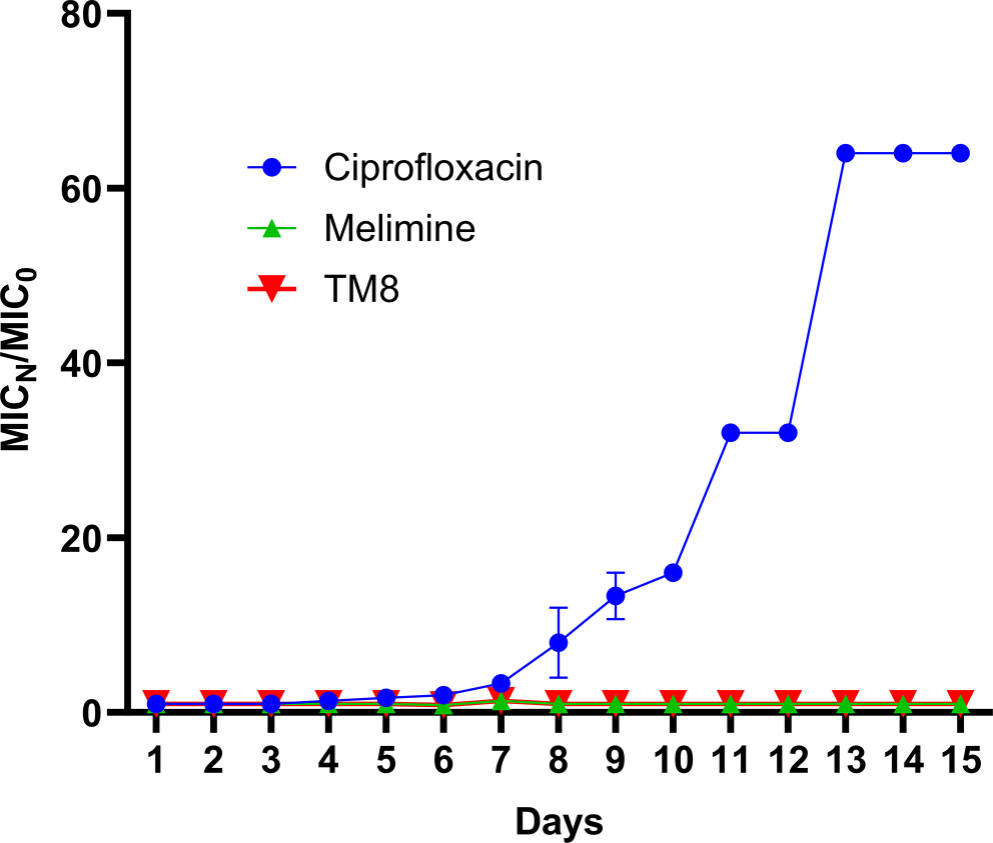
Fold-change in MIC of antimicrobials during 15-day bacterial passage of *E. cloacae* 008 in the presence of different antimicrobials at sub-MICs. MIC_N_ = MIC on day N after first growth; MIC_0_ = MIC on the first day of growth at sub-MIC. Data represent mean ± SEM from three independent biological replicates, each performed in triplicate. Statistical analysis was performed using two-way ANOVA with Greenhouse-Geisser correction (ε = 0.5) followed by Dunnett’s post hoc test. Significant effects were detected for treatment (p<0.000001), time (p<0.000001), and their interaction (p<0.000001). A marked increase in MIC was observed for ciprofloxacin from day 6 onward, whereas melimine and TM8 remained stable throughout the 15-day period (adjusted p = 0.000007).

As *E. cloacae* had not previously been tested for development of resistance to peptoids, the potential for polymyxin-resistant *E. cloacae* 008 to develop resistance to TM8 was assessed by exposing the bacterium to sub-MICs (0.5 ×) of the compound over fifteen consecutive days. The experiment was performed in parallel with melimine and ciprofloxacin at their respective sub-MICs. The MIC of ciprofloxacin started to increase on day six rising from 0.25 µg mL^−1^ to 0.5 µg mL^−1^. On day 11, the MIC reached 8 µg mL^−1^, and from day 13 to 15, it reached and remained at 64 µg mL^−1^. In contrast, the MICs for both melimine and TM8 remained constant throughout the 15-day period (Fig. 3). There was a significant difference in resistance development when comparing ciprofloxacin with TM8 or melimine.

### Evaluation of combinations of TM8 and antibiotics in polymyxin-resistant bacteria

All polymyxin-resistant isolates were tested for synergy between colistin and TM8. Rifampicin, although clinically ineffective against Gram-negative bacteria due to outer membrane impermeability (Mukherjee et al. 2024), was also tested with TM8 based on its reported synergy with peptidomimetics (Mood et al. 2021). Selection of isolates for combination testing included both resistant or intermediate strains as classified by CLSI guidelines (CLSI 2025). A ciprofloxacin-sensitive, polymyxin-resistant *E. cloacae* clinical isolate was chosen to assess TM8’s activity in a strain otherwise sensitive to fluoroquinolones. TM8 exhibited synergy with rifampicin and ciprofloxacin in 66.7% of tested cases (2 out of 3). When TM8 was used in tandem with colistin, an additive effect was observed in 66.7% (4 out of 6) of cases, while the remaining two cases showed synergy in one and indifference in the another (Table 6).

**Table 6:** Checkerboard assay results of TM8 with different antibiotics against different polymyxin-resistant Gram-negative bacteria.

Isolates	Antibiotic	MIC of TM8 (µg mL^−1^)	MIC of TM8 + antibiotic (µg mL^−1^)	FIC_TM8_	MIC of antibiotic (µg mL^−1^)	MIC of antibiotic + TM8 (µµg mL^−1^)	FIC_Antibiotic_	ƩFIC	Outcome	Fold reduction in TM8’s MIC	Fold reduction in antibiotic’s MIC
***A. baumannii* 19606-R**	Colistin	7.8	3.9	0.5	256	128	0.5	1	Additive	2	2
	Minocycline	7.8	3.9	0.5	4	2	0.5	1	Additive	2	2
	Ciprofloxacin	7.8	3.9	0.5	256	128	0.5	1	Additive	2	2
	Rifampicin	7.8	3.9	0.5	32	16	0.5	1	Additive	2	2
	Gentamicin	7.8	7.8	1	256	256	1	2	Indifference	1	1
***E. cloacae* 008**	Colistin	15.6	3.9	0.25	32	1	0.03	0.28	Synergy	4	32
	Minocycline	15.6	7.8	0.5	8	2	0.25	0.75	Additive	2	4
	Ciprofloxacin	15.6	1.95	0.125	0.25	0.0312	0.125	0.25	Synergy	8	8
***E. coli* NCTC13846**	Colistin	7.8	3.9	0.5	8	4	0.5	1	Additive	2	2
	Minocycline	7.8	7.8	1	8	8	1	2	Indifference	1	1
	Rifampicin	7.8	1.95	0.25	16	0.0312	0.0018	0.2518	Synergy	4	8
***K. pneumoniae* 13883-R**	Colistin	62.5	62.5	1	256	128	0.5	1.5	Indifference	1	2
	Minocycline	62.5	62.5	1	2	0.25	0.125	1.125	Indifference	1	2
	Rifampicin	62.5	15.6	0.25	16	0.25	0.015	0.265	Synergy	4	64
**PAO1-R**	Colistin	31.2	15.6	0.5	128	32	0.25	0.75	Additive	2	4
***P. aeruginosa* 216**	Colistin	7.8	3.9	0.5	16	8	0.5	1	Additive	2	2
	Ciprofloxacin	7.8	1.95	0.25	0.5	0.0625	0.125	0.375	Synergy	4	8

### Antibacterial activity of TM8 in the presence of different salts

As AMPs can lose activity in high salt, with 2–3-fold reductions reported for NaCl and MgCl_2_ (Tomita et al. 2000), their effects on peptoid TM8 was studied. Monovalent salts had minimal impact (≤2-fold MIC change), while divalent cations (MgCl_2_, CaCl_2_) caused a 2–4-fold MIC increase across all strains (Table 7).

**Table 7:** Fold-change in MIC of TM8 against bacteria under different salt exposures.

Strains	Fold-change in MIC upon treatment with				
	NaCl (150 mM)	KCl (4.5 mM)	NH_4_Cl (6 μM)	MgCl_2_ (1 mM)	CaCl_2_ (2.5 μM)
***A. baumannii* ATCC 19606**	2	1	1	2	2
***A. baumannii* 19606-R**	1	2	2	2	2
***K. pneumoniae* ATCC 13883**	2	2	1	4	2
***K. pneumoniae* 13883-R**	2	2	2	2	4
**PAO1**	1	1	1	4	4
**PAO1-R**	2	2	1	2	4

## Discussion

Peptoids have shown promising antimicrobial activity against ESKAPEE pathogens, with TM8 effective against MBL-producing, polymyxin-sensitive, extensively drug-resistant (XDR) bacteria (Mishra et al. 2025b), prompting evaluation against polymyxin-resistant Gram-negatives. As polymyxins are last-resort antibiotics for managing XDR infections caused by Gram-negative ‘superbugs’ (Velkov et al. 2016, Yang et al. 2020), new therapeutic alternatives are urgently needed.

In *A. baumannii*, loss of lipid A or LPS is the main mechanism of polymyxin resistance, as seen in ATCC 19606-R and 13 colistin-resistant derivatives carrying mutations in lipid A biosynthesis genes (*lpxA, lpxC*, and *lpxD*) (Moffatt et al. 2010). In *E. coli* NCTC 13846, resistance is mediated by *mcr-1*, encoding a phosphoethanolamine-lipid A transferase that reduces the overall negative charge of LPS (Shahzad et al. 2023).

Whole genome sequences (WGSs) of *K. pneumoniae* 13883-R and *P. aeruginosa* PAO1-R were performed as the basis for their polymyxin resistance had not previously been reported. In *K. pneumoniae*, apart from capsule formation, two-component system (TCS)-mediated OM remodelling (*phoP, phoQ, pmrA, pmrB, pmrD, crrA, crrB, crrC*, and *mgrB*), efflux pumps and outer membrane proteins (OMPs), *mcr*-mediated resistance, modification of lipid A involving *arnBCADTEF* operon, *pmrE, pmrC, lpxM*, and *yciM* genes have been reported to confer polymyxin resistance (Formosa et al. 2015, Halaby et al. 2016, Elias et al. 2021, Mirshekar et al. 2024, Padhy et al. 2024). A previous proteomic study of *K. pneumoniae* 13883-R revealed an enrichment of OMPs associated with stress response, glutamine degradation, and the metabolic pathways of pyruvate, aspartate, and asparagine (Jasim et al. 2018).

In the current WGS analysis of *K. pneumoniae* 13883-R, high-impact mutations were identified in *evgA, gltP, ccrB, lptD*, and *mlaB*, alongside 55 other genes. *evgA* is a regulator gene (Singh et al. 2025) and is involved in increasing drug resistance in Gram-negative bacteria (Altayb et al. 2022). In this study, the truncation mutation in *evgA* (Tyr87*) is predicted to inactivate the EvgSA regulatory system as it removes the C-terminal DNA binding domain. This may contribute to altered membrane physiology, but is unlikely to contribute directly to polymyxin resistance. GltP is a proton-coupled transporter, and mutations (deletion, frameshift) in *gltP* have been associated with altered membrane homeostasis contributing to AMP tolerance, potentially through effects on lipid composition or membrane asymmetry (Sandin et al. 2022). In the current study with respect to polymyxin-resistant *K. pneumoniae* isolate, the Gln437* stop-gained mutation in *gltP* is predicted to result in envelope adaptations that support colistin resistance. CcrB is associated with resistance to colistin (Sanchez-Leon et al. 2023) via lipid A modification with 4-amino-4-deoxy-L-arabinose (L-Ara4N) and palmitoylation (Sun et al. 2020). In the mutant *K. pneumoniae* 13883-R isolate, the Tyr241* stop-gained mutation likely disrupted a component of the lipid A modification pathway linked to polymyxin resistance. In this isolate, the mutation could have contributed to a broader change in lipid A remodeling, though its precise effect remains unclear. A previous study on *K. pneumoniae* mutants evolved through serial passaging in the presence of colistin also reported mutations at various nucleotide positions within *ccrB* (T88C, C434T, or C9665T) as well as at other positions (T398G, T585A, or G976A) (Aulin et al. 2021). Similarly, another study on a colistin-resistant mutant of *K. pneumoniae* ATCC BAA 2146 identified a *ccrB* missense mutation encoding the HAMP domain-containing histidine kinase CrrB, together with a silent *satP* mutation (Sun et al. 2020), suggesting possible alterations in two-component regulatory signaling. MlaB is a cytoplasmic component of an ABC transporter, MlaFEDB, with possible regulatory function (Kolich et al. 2020), which maintains outer membrane integrity by trafficking phospholipid across the bacterial envelope (Liu et al. 2021, Sun et al. 2022). The Ser96* stop-gained mutation in *mlaB* is predicted to inactivate this component of the MlaFEDB complex. The loss of MlaB perhaps contributes to a remodeled outer membrane state associated with polymyxin resistance. LptD, which contains a periplasmic N-terminal domain and a transmembrane C-terminal β-barrel, is a core component of the LptABCDEFG machinery that transports LPS across the outer leaflet of the outer membrane (Qiao et al. 2014, Botos et al. 2016). The Ser45Gly missense mutation in *lptD* may disrupt LPS assembly or outer membrane integrity, potentially altering surface charge or permeability. Such change could contribute to the polymyxin resistance observed in *K. pneumoniae* 13883-R strain. Overall, the changes in genes seen with *K. pneumoniae* 13883-R suggest changes to membranes in this strain, some or all of which might be involved in polymyxin resistance, but these need to be experimentally determined.

In *P. aeruginosa*, polymyxin resistance can arise through multiple mechanisms, including outer membrane remodelling involving *phoP, phoQ, pmrA, pmrB, pmrR, pmrS, colR, colS, cprR, cprS*, as well as LPS modification-independent mechanisms, involving *pdxB*-mediated metabolism, *oprH* overexpression, efflux pumps, acquired *mcr* genes, and *parRS*-mediated LPS modifications (Bell et al. 1991, Fernández et al. 2010, Lee et al. 2014, Snesrud et al. 2018, Lorusso et al. 2022, Bakleh et al. 2024, Padhy et al. 2024). In the current study, seven genes, *pmrA, mexT, masA, napA*, PA1327, PA0683, and PA2141, were observed in the mutant strain. Previous studies have reported that *pmrA* SNPs that drive *arnBCADTEF* operon overexpression, leading to L-Ara4N addition to lipid A, and reduced polymyxin binding (Gunn et al. 1998, McPhee et al. 2006). In the current study, despite having a frameshift mutation in *pmrA* that is predicted to cause its truncation, the PAO1-R strain exhibited polymyxin resistance. We hypothesize that either the truncated PmrA protein retains deregulated activity or that compensatory changes in parallel envelope stress pathways drive lipid A modification independently of PmrA. In this context, the mexT frameshift, which is expected to alter global regulation and efflux, likely contributes indirectly to the resistant phenotype by remodelling the cell surface and stress responses. The genes *masA, napA*, PA0683, PA1327, and PA2141 have not been explored previously for a role in polymyxin resistance.

Comparative WGS of *P. aeruginosa* 216 (Khan et al. 2020) showed resistance determinants distinct from PAO1-R, including *arnA* mutations involved in the addition of L-Ara4N to LPS (Gatzeva-Topalova et al. 2005), as well as in genes potentially affecting MexAB-OprM efflux pump (Braz et al. 2016). This strain possesses *speD2* (Damtie et al. 2025), which is essential for spermidine biosynthesis on the bacterial surface, where spermidine contributes to protection of the *P. aeruginosa* outer membrane against polymyxin-mediated disruption (Johnson et al. 2012, Han et al. 2018). However, functional or expression analyses were not performed to confirm the role of *speD2* in the isolate. There was no evidence for the *mcr*-acquired genes in either *P. aeruginosa* or *K. pneumoniae* isolates based on ResFinder analysis.

The peptoids used in this study were derived from the TM1, a dodecamer of lysine-like, cationic monomers (*N*Lys) and phenylalanine-like hydrophobic, aromatic monomers (*N*spe) (Czyzewski et al. 2016). The tetra-brominated peptoid TM4 showed enhanced antimicrobial activity by 2–16-fold compared to its di-brominated short analogue TM2 with the largest difference in activity against *E. cloacae* and *A. baumannii* 19606-R. This aligns with the previous findings that bromination can increase the activity of peptoids (Molchanova et al. 2020). The C-terminal *N*Lys difference between TM2 and TM4 may also have contributed to the beneficial effects against PAO1-paired strains and *E. coli*. Overall, the peptoid that showed the lowest geometric mean MIC considering all the polymyxin B-resistant isolates tested was TM8 (15.6 µg/mL), in line with previous reports (Nielsen et al. 2022). TM8 also reduced *P. aeruginosa* biofilm viability more effectively than TM1 or TM6 (Nielsen et al. 2022). Self-assembly in lipopeptoids can occur by covalent attachment of the lipophilic tail domains forming micellar macromolecules reinforced by the intermolecular hydrophobic interactions (Lau et al. 2017). TM8 self-assembles into an ellipsoid making strong intermolecular interactions (Nielsen et al. 2022). Therefore, supramolecular assembly may be at least partly responsible for its activity (Wardell et al. 2025).

Activity of TM8 was also investigated using flow cytometry and LIVE/DEAD staining with the *A. baumannii* pairing. The greatest difference occurred at 4 × MIC, where there were more cells stained with PI in the resistant mutant compared to the parent strain. This may reflect the known compromises to the outer membrane of the resistant strain, and easier access of the peptoid to the inner membrane of the bacterial cell causing lysis (Moffatt et al. 2010).

While the exact mechanism of polymyxin resistance in *E. cloacae* 008 has yet to be studied, the current study examined its ability to develop resistance to TM8 upon exposure to sub-MIC concentrations. The organism developed a 64-fold rise in ciprofloxacin MIC; however, no resistance was observed for TM8 suggesting limited potential for resistance development under the tested conditions. The inability of polymyxin-resistant bacteria to develop resistance to TM8 replicates a previous study in which *P. aeruginosa* strain did not develop resistance to TM5, a 5mer peptoid structurally related to TM8 but with a longer tridecyl (C13 tail) and two fewer *N*spe moieties compared to TM8. However, that strain exhibited a 4-fold increase in gentamicin MIC after only five days of serial passage (Lin et al. 2022). In the current study, the bacteria did not develop resistance towards melimine as well. This has been previously demonstrated for *Staphylococcus aureus* and *P. aeruginosa* (Yasir et al. 2020, 2021). However, under similar laboratory conditions, resistance to polymyxin by ESKAPEE pathogens can occur (Spohn et al. 2019). This reinforces the concept that AMPs and peptoids act through modes of action distinct from polymyxin antibiotics, at least with respect to the isolates and resistance mechanisms identified in this study.

To explore this further, the current study examined whether there was synergistic activity of the TM8 against polymyxin-resistant bacteria in combination with colistin, minocycline, rifampicin, ciprofloxacin, or gentamicin. Notably, colistin, rifampicin, and ciprofloxacin showed synergistic effects. In *E. cloacae*, TM8-colistin synergy reduced colistin MIC below the clinical breakpoint. Similarly, TM8-rifampicin synergy lowered rifampicin MIC, which was analyzed considering *S. aureus* breakpoint, as no CLSI breakpoints exist for rifampicin against *E. coli, K. pneumoniae*, or other Gram-negative bacteria (CLSI 2025). Given that colistin may exhibit synergism with protein and RNA synthesis inhibitors in *mcr*-mediated colistin resistance, combination therapy remains a valuable strategy (Brennan-Krohn et al. 2018, Li et al. 2018). While combination therapy of antibiotics is a promising strategy to curb the development of resistance, it also carries the risk of imposing selection pressure for resistance to multiple antibiotics at once, thereby, potentially inducing resistance evolution (Siedentop et al. 2024). On the contrary, bacteria are refractory to developing resistance to AMPs and their mimetics. Therefore, combining peptoids with antibiotics at synergistic concentrations presents more a justifiable alternative compared to traditional antibiotic-antibiotic combinations.

As polymyxin resistance mechanisms may overlap with those affecting AMPs (Trimble et al. 2016, Tajer et al. 2024), it was hypothesized that resistance to polymyxin might confer cross-resistance to peptoids. However, this was not typically observed in this study for TM8, as the differences in MIC between polymyxin-sensitive and -resistant Gram-negative bacterial pairs were typically ≤ 2-fold, suggesting a distinct or multifaceted mode of action. The main site of action of polymyxins is the outer membrane of Gram-negative bacteria (Poirel et al. 2017, Akhoundsadegh et al. 2019). While AMPs can also act on the outer membrane, they also act on the inner membrane and peptidoglycan (Talapko et al. 2022), and peptoids have similar modes of action to AMPs (Mojsoska et al. 2017). For instance, TM1 has been shown to induce intracellular biomass flocculation besides having membrane permeabilizing effects (Chongsiriwatana et al. 2017).

While AMPs face challenges such as high production cost, proteolytic instability, and salt sensitivity (Tomita et al. 2000, Tan et al. 2021, Cashman-Kadri et al. 2023), peptoids resist protease degradation (Sara et al. 2024). In the current study, TM8’s activity was not found to be appreciably impacted by the monovalent cations. However, the divalent salts, MgCl_2_ at physiological concentration and CaCl_2_ at sub-physiological concentration, had some inhibitory effect on its activity.

## Conclusions

Based on MIC determination, TM8 emerged as the most effective candidate against all tested polymyxin-resistant bacteria. Importantly, no resistance development was observed against TM8. It demonstrated synergistic activity with antibiotics from different classes and showed similar MICs against both polymyxin-sensitive and polymyxin-resistant paired strains of Gram-negative bacteria highlighting its diverse mode of action from polymyxins. These attributes highlight its potential as a promising antibacterial agent against polymyxin-resistant infections. Nonetheless, further studies involving a larger collection of colistin-resistant isolates, along with *in vivo* assessment of TM8’s efficacy and safety, are warranted to advance its clinical development.

### Limitations

This study primarily focused on non-*mcr*-mediated polymyxin resistance. Accordingly, a detailed investigation of *mcr*-1-harboring strains was not undertaken; only a reference *E. coli* strain carrying *mc*r-1 was included. Moreover, no paired isolates carrying both *mcr* and non-*mcr* determinants were analyzed. Instead, three representative laboratory-derived polymyxin-sensitive and -resistant isolate pairs, along with two clinical isolates, were examined. Likewise, not all strains underwent WGS. Attending to these limitations in future studies may offer new insights into the interplay between polymyxin and AMP resistance mechanisms.

## Supplementary Material

lxag093_Supplemental_Files

## Data Availability

Figure S1: Heatmap of MIC fold-change in MIC across wild-type and polymyxin-resistant mutants of *A. baumannii, K. pneumoniae* and *P. aeruginosa* when treated with melimine, Mel4 and a panel of peptoids. Figure S2: Schematic representation of genes associated with polymyxin resistance in *K. pneumoniae* and *P. aeruginosa* isolates AMR profiles of KP: Table S1 Acquired AMR genes_ResFinder_KP-R; Table S2 AMR genes-CARD database-KP-R AMR profiles of KP: Table S3 Acquired AMR genes-Resfinder_PA-R; Table S4 AMR genes-CARD database-PA-R Table S5 High impact variant of KP-R detected by SnpEff Table S6 High impact variant of PA-R detected by SnpEff The nucleotide sequences of *K. pneumoniae* 13883-R (SAMN48355430) and *P. aeruginosa* PAO1-R (SAMN48355431) used in this study have been deposited in GenBank under the BioProject accession number PRJNA1259179.
