# Supplementary figures and images for: Peptoid-based antimicrobial strategies against polymyxin-resistant Gram-negative bacteria
Source: J Appl Microbiol. 2026 Apr 11;137(4):lxag093. doi: 10.1093/jambio/lxag093 (PMC13116114; doi:10.1093/jambio/lxag093)

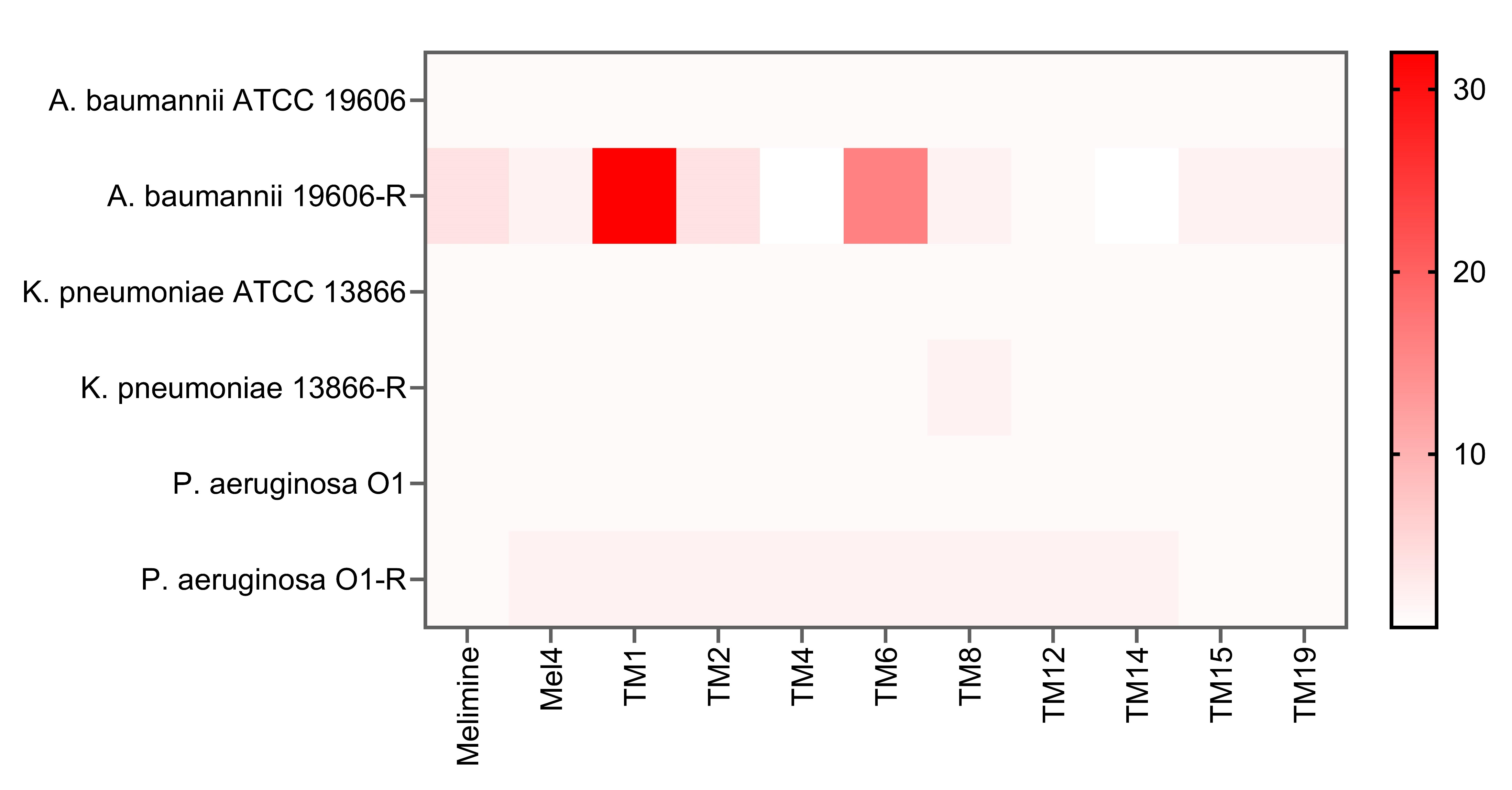

Supplement: lxag093_Supplemental_Files [file lxag093_supplemental_files.zip › Figure S1 Heatmap of MIC fold-change across wild-type and polymyxin-resistant mutants.tif]

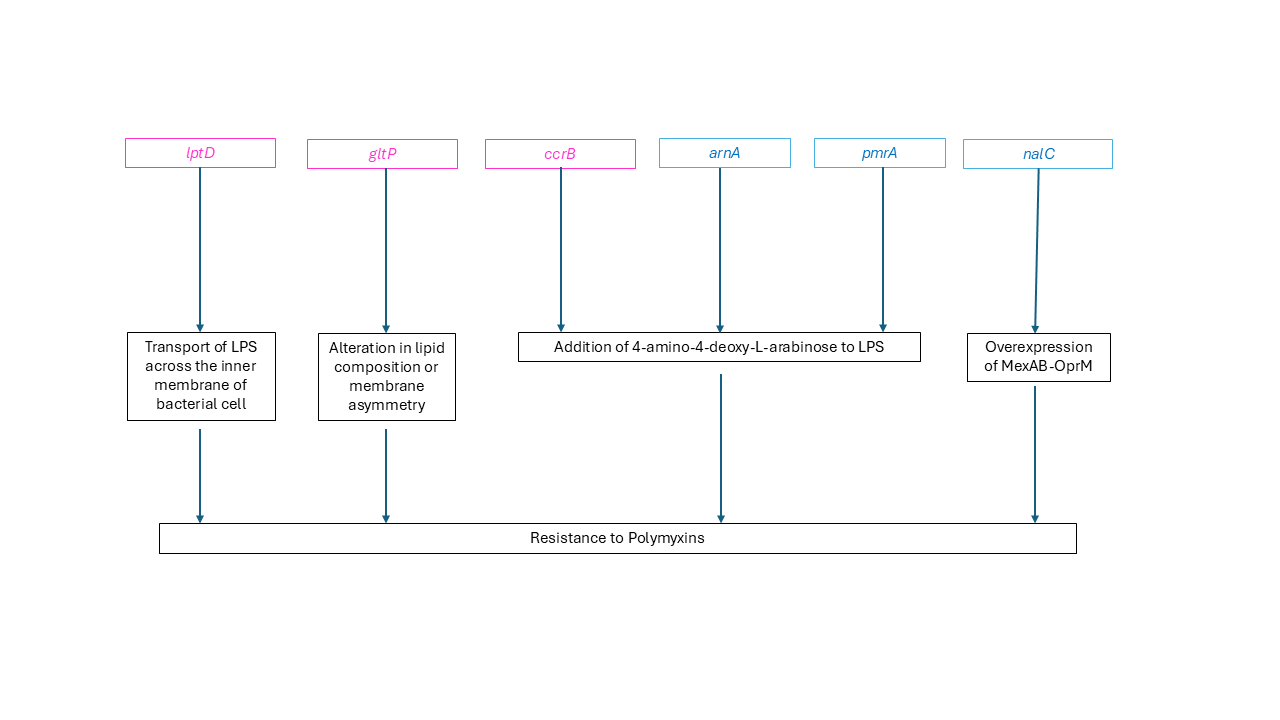

Supplement: lxag093_Supplemental_Files [file lxag093_supplemental_files.zip › Figure S2 Schematic diagram showing genes associated with Polymyxin resistance.tif]
